# Hyperparathyroidism Secondary to Burosumab Treatment

**DOI:** 10.7759/cureus.89569

**Published:** 2025-08-07

**Authors:** Minhtri K Nguyen, Dhiresh Bandaru, Minh-Kevin Nguyen

**Affiliations:** 1 Medicine, Ronald Reagan University of California Los Angeles Medical Center, Los Angeles, USA; 2 Medicine, University of California, Riverside School of Medicine, Riverside, USA; 3 Medicine, Western University of Health Sciences, Pomona, USA

**Keywords:** burosumab, fgf-23, hyperparathyroidism, hypophosphatemia, tumor induced osteomalacia

## Abstract

Tumor-induced osteomalacia (TIO) is a rare paraneoplastic syndrome of abnormal phosphorus metabolism caused by increased secretion of fibroblast growth factor 23 (FGF23) by small mesenchymal tumors. In this article, we reported a patient with chronic refractory hypophosphatemia due to TIO who is treated with burosumab, a monoclonal antibody that targets and blocks the activity of FGF23. Treatment with burosumab led to the resolution of his refractory hypophosphatemia, but this was complicated by the development of secondary hyperparathyroidism.

This article reports a rare case of hyperparathyroidism induced by burosumab in a patient with TIO.

## Introduction

Tumor-induced osteomalacia (TIO) is a rare paraneoplastic syndrome characterized by abnormal phosphorus metabolism, resulting from the secretion of fibroblast growth factor 23 (FGF23) by small mesenchymal tumors [1-5]. The impaired bone metabolism in TIO is due to FGF23-induced hypophosphatemia resulting from renal phosphate wasting as well as decreased intestinal phosphate absorption through decreased 1,25-dihydroxy vitamin D production. Previously, we reported a refractory case of chronic hypophosphatemia attributed to TIO after an extensive investigation [6]. Our patient was found to have a hyperdense lesion in C7-T1 on PET/CT scan that was concerning for a mesenchymal tumor. Since he was deemed not a surgical candidate based on his tumor location, our patient was treated with burosumab, which is a monoclonal antibody that targets and blocks the activity of FGF23 [4,6]. Treatment with burosumab led to the resolution of his refractory hypophosphatemia, but this was complicated by the development of secondary hyperparathyroidism. To date, there is very limited data on burosumab-induced hyperparathyroidism. Previously, Zhukouskaya et al. reported the development of hyperparathyroidism after three years of burosumab in children affected with X-linked hypophosphatemia [7]. However, hyperparathyroidism secondary to burosumab has not been reported in patients with tumor-induced osteomalacia. In this article, we report a rare case of hyperparathyroidism due to burosumab treatment in a patient with tumor-induced osteomalacia.

## Case presentation

A 71-year-old man with a past medical history significant for atrial fibrillation, monoclonal gammopathy of unclear significance (MGUS), and osteoporosis presented with progressive muscle weakness and bony pain [6]. He was previously diagnosed with debilitating osteoporosis with multiple pathologic fractures in the axial and appendicular skeleton (multiple rib fractures and bilateral hip fractures), and a past bone marrow biopsy was normal [6]. Laboratory evaluation revealed severe hypophosphatemia, inappropriately low normal 1,25-dihydroxy vitamin D despite being on cholecalciferol and calcitriol, normal 25-hydroxy vitamin D, normal calcium level, normal serum albumin, normal serum creatinine, low 24-hour urinary calcium excretion, and normal parathyroid hormone (PTH) (Table 1).

**Table 1:** Laboratory parameters *Expected value in the setting of hypophosphatemia if the etiology is extrarenal. PTH: parathyroid hormone; FEPO4: fractional excretion of phosphate; FGF23: fibroblast growth factor 23

Test	Result	Reference range
Phosphorus	1.7 mg/dL	2.5-4.5 mg/dL
1,25-diOH vitamin D	34.3 pg/mL	19.9-79.3 pg/mL
25-OH vitamin D	47 ng/mL	20-50 ng/mL
Calcium	9.9 mg/dL	8.6-10.4 mg/dL
Albumin	4.6 g/dL	3.9-5.0 g/dL
Creatinine	1.1 mg/dL	0.6-1.3 mg/dL
24-hour urine calcium	23 mg	55-300 mg/24 hr
Intact PTH	42 pg/mL	11-51 pg/mL
FEPO4	20.40%	<5% *
24-hour urine phosphate	739.8 mg	<100 mg/24 hr *
FGF23	321 RU/mL	<180 RU/mL

**Figure 1:**
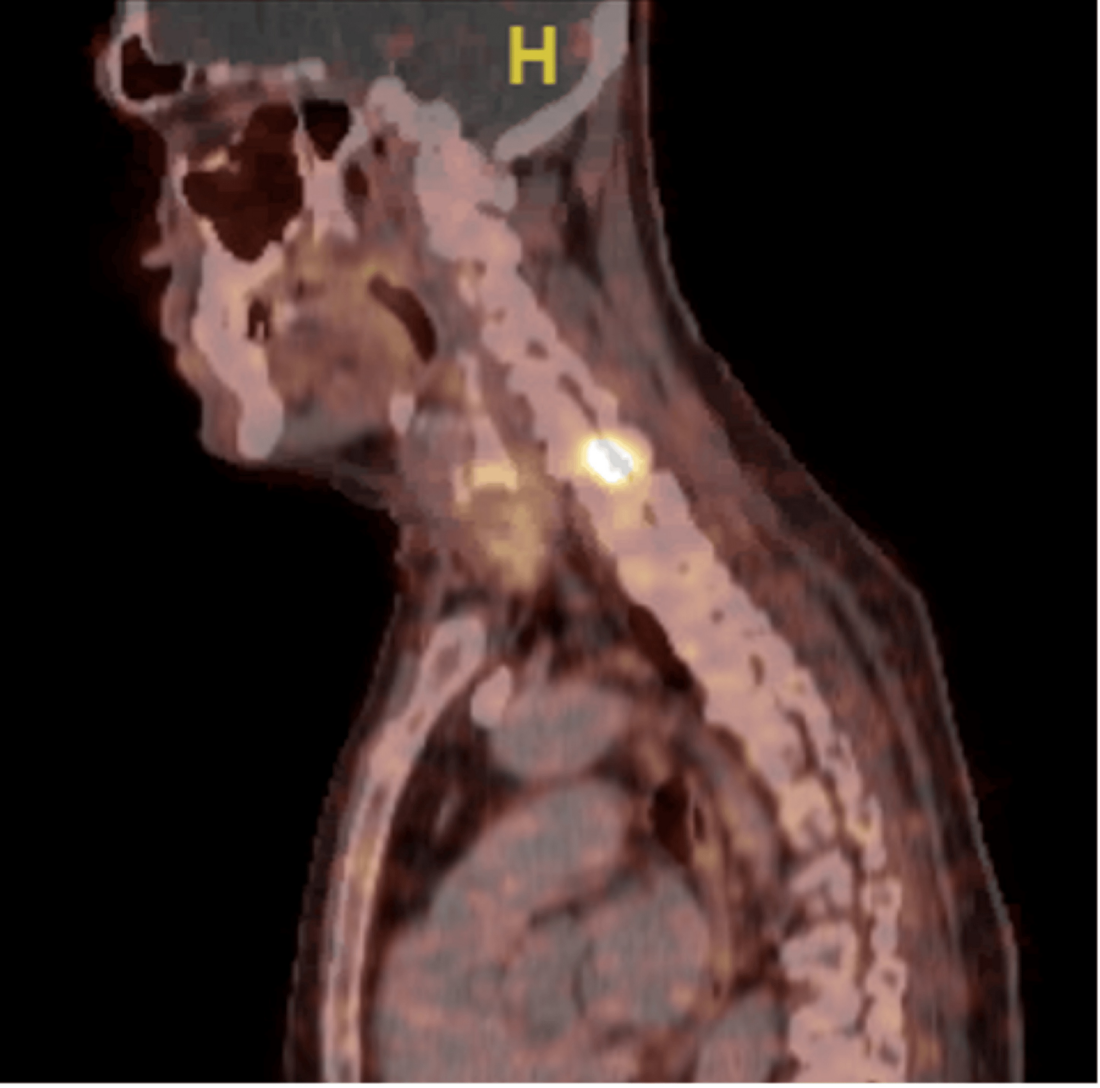
Hyperdense lesion in C7-T1 concerning for a mesenchymal tumor Radiographic imaging via 68Ga-DOTATATE-based positron emission tomography (PET)/computed tomography (CT) revealed a hyperdense lesion in C7-T1 concerning for a mesenchymal tumor.

Subsequent urinary testing showed high fractional excretion of phosphate (FEPO4) and inappropriate renal phosphate wasting on 24-hour urinary collection (Table 1). Fanconi syndrome was excluded, given the absence of glucosuria and non-gap metabolic acidosis. Further serum testing for fibroblast growth factor 23 (FGF23) was remarkably high (Table 1). Follow-up radiographic imaging via 68Ga-DOTATATE-based positron emission tomography (PET)/computed tomography (CT) revealed a hyperdense lesion in C7-T1 concerning for a mesenchymal tumor (Figure 1) [6].

**Figure 2:**
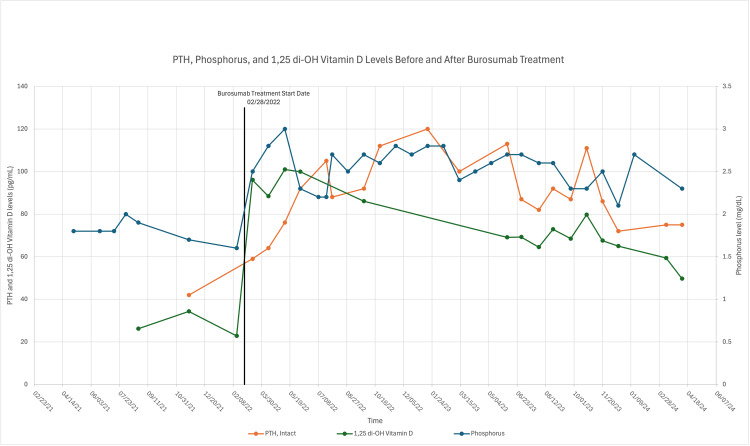
Effect of burosumab on PTH, phosphorus and 1,25-dihydroxy vitamin D levels The figure reveals parathyroid hormone (PTH), phosphorus, and 1,25-dihydroxy vitamin D levels before and after burosumab treatment.

The hyperdense lesion in C7-T1 was subsequently confirmed on cervical spine MRI. He was deemed not to be a surgical candidate based on the tumor location, and he was treated with anti-FGF23 therapy with subcutaneous burosumab monthly [6]. Of note, his severe hypophosphatemia was refractory to phosphate repletion and cholecalciferol and calcitriol supplementation. Treatment with subcutaneous burosumab resulted in normalization of serum phosphate levels and resolution of his musculoskeletal symptoms. Follow-up laboratory tests after initiation of burosumab therapy revealed an increase in 1,25-dihydroxy vitamin D and PTH levels (Figure 2).

## Discussion

We report a case of a 71-year-old male who developed debilitating bony pain, pathologic fractures, and progressive muscle weakness. Laboratory evaluation was notable for severe hypophosphatemia and inappropriately low normal 1,25-dihydroxy vitamin D level despite being treated with cholecalciferol and calcitriol, thereby prompting additional investigation. Further testing revealed that the phosphaturia was due to markedly elevated FGF23. His imaging studies identified a C7-T1 hyperdense lesion consistent with a mesenchymal tumor.

Tumor-induced osteomalacia (TIO) is a rare clinical disorder characterized by renal phosphate wasting due to the ectopic secretion of FGF23 and is typically caused by a mesenchymal tumor [1-5]. In our patient, the mesenchymal tumor is localized to the C7-T1 spine and is likely the source of the excessive secretion of FGF23 [6]. FGF23 is a circulating peptide that plays a critical role in maintaining serum phosphate levels [5,8]. It is produced by bone cells, specifically osteocytes and osteoblasts, in response to increased dietary phosphate intake [8].

FGF23 helps regulate the serum phosphate level by decreasing phosphate reabsorption in the kidneys and lowering phosphate absorption in the intestines [5,9]. In the proximal tubules of the kidney, FGF23 binds to its receptor and coreceptor klotho, leading to the inhibition of the sodium-phosphate cotransporter Na/Pi IIa on the luminal membrane [5,9]. This action reduces phosphate reabsorption and increases its excretion in urine. Additionally, FGF23 downregulates the expression of the enzyme 1-alpha-hydroxylase in proximal tubular cells, thereby reducing calcitriol synthesis in the kidneys and subsequently decreasing intestinal phosphate absorption [5,9]. Consequently, TIO should be suspected in patients with progressive weakness, bone pain, multiple fractures due to osteomalacia, and hypophosphatemia in the setting of phosphaturia and low or inappropriately normal 1,25-dihydroxy vitamin D level. In our patient, the high fractional excretion of phosphate (20.4%) and inappropriate renal phosphate wasting (740 mg/24hr) in the setting of severe hypophosphatemia is consistent with the renal phosphate wasting effect of FGF23. Moreover, the inappropriately low 1,25-dihydroxy vitamin D in the setting of significant hypophosphatemia is reflective of the inhibitory effect of FGF23 on the proximal tubular expression of 1-alpha-hydroxylase enzyme.

**Figure 3:**
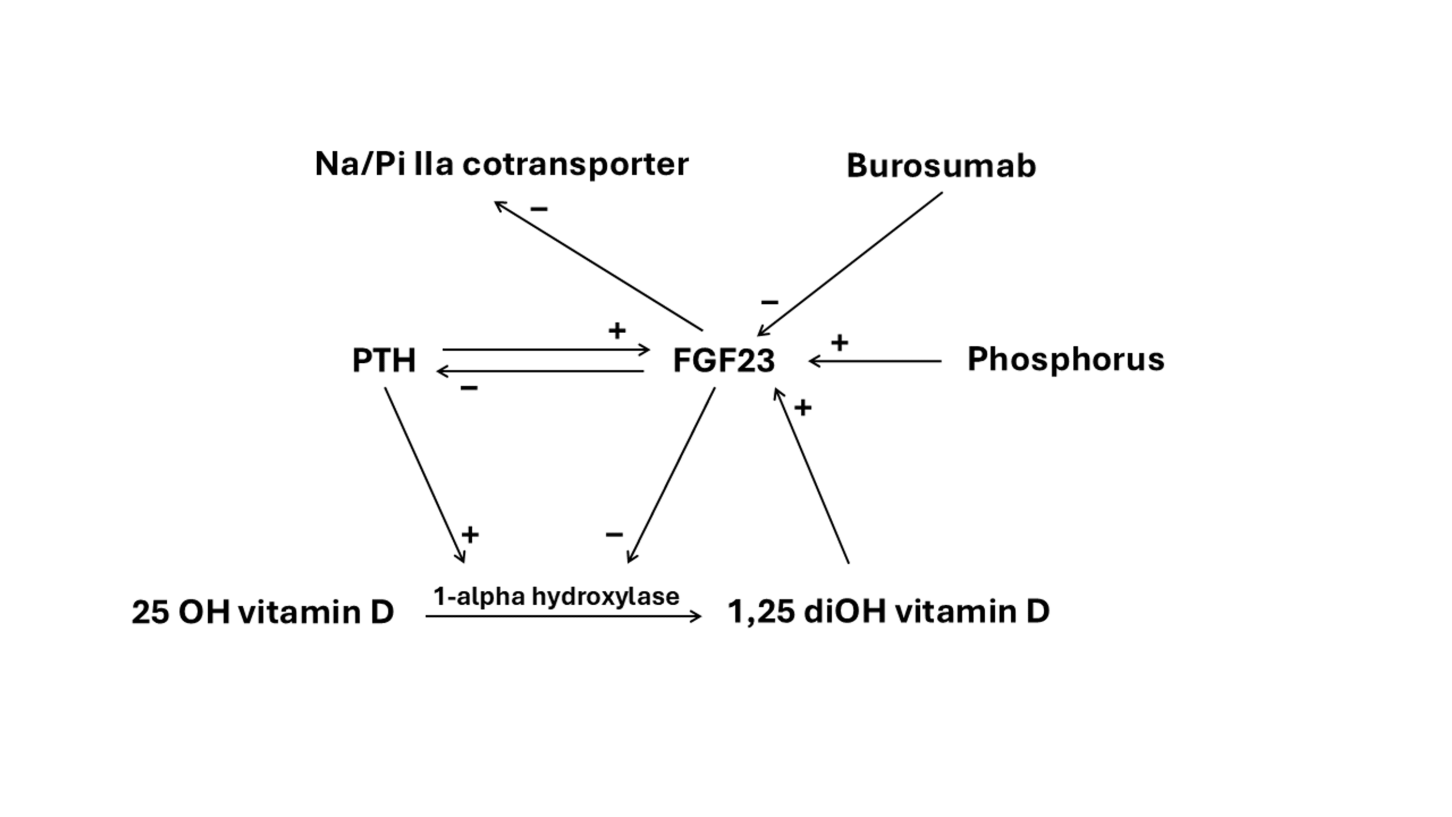
Role of FGF23 in the pathogenesis of TIO FGF23 is secreted in response to increased dietary phosphate load, 1,25-dihydroxy vitamin D, and PTH. FGF23 induces renal phosphate wasting by inhibiting the sodium phosphate cotransporter Na/Pi IIa in the renal proximal tubule, and it reduces intestinal phosphate absorption by inhibiting the 1-alpha hydroxylase enzyme, resulting in decreased 1,25-dihydroxy vitamin D synthesis by the kidney. Since PTH secretion is suppressed by FGF23, burosumab inhibits the inhibitory effect of FGF23 on PTH, thereby resulting in secondary hyperparathyroidism. FGF23: fibroblast growth factor 23; TIO: tumor-induced osteomalacia; PTH: parathyroid hormone

Curative surgical intervention is the preferred treatment for tumor-induced osteomalacia. However, our patient was deemed not to be a surgical candidate based on the tumor location, given the higher risk of neurological complications from surgical intervention [6]. Therefore, our patient was initiated on anti-FGF23 therapy with subcutaneous burosumab monthly. Burosumab is a human monoclonal antibody to FGF23 [4]. After a few months of subcutaneous burosumab therapy, our patient's symptoms significantly improved with normalization of the serum phosphorus concentration. Follow-up laboratory tests after the initiation of burosumab therapy revealed an increase in 1,25-dihydroxy vitamin D and PTH levels (Figure 2). The increase in 1,25-dihydroxy vitamin D level and intact PTH level was likely due to the underlying mechanism of action of burosumab. Since burosumab inhibits the inhibitory effect of FGF23 on the renal 1-alpha-hydroxylase enzyme, it is not surprising that there is an increase in the 1,25-dihydroxy vitamin D level post-treatment. Interestingly, there is also an increase in PTH levels after treatment with burosumab. Of note, the patient’s serum creatinine remains normal during this time; serum creatinine was 1.01 mg/dL with estimated glomerular filtration rate (eGFR) of 79 ml/min/1.73 m2 when PTH was at its peak level of 120 pg/mL. In a recent abstract, Zhukouskaya et al. reported the development of hyperparathyroidism after three years of burosumab in children affected with X-linked hypophosphatemia whose baseline PTH levels were normal prior to therapy [7]. The mechanism of the hyperparathyroidism induced by burosumab was unclear. In this case report, we reported a case of hyperparathyroidism secondary to burosumab in a patient with tumor-induced osteomalacia with a normal baseline PTH level. In a study on rats, both PTH expression and secretion were suppressed by FGF23, making the parathyroid gland one of its targets [10]. Since burosumab inhibits the inhibitory effect of FGF23 on PTH, burosumab may result in increased PTH secretion and the development of secondary hyperparathyroidism. Figure 3 shows the role of FGF23 in the pathogenesis of TIO.

However, there are conflicting data on the effect of FGF23 on PTH secretion. Using both rats and in vitro rat parathyroid cultures, Ben-Dov et al. demonstrated that FGF23 suppressed both parathyroid hormone secretion and PTH gene expression [10]. In contrast, in a mouse model of chronic kidney disease, Kawakami et al. showed that FGF23 increased both parathyroid cell proliferation and PTH release, and both effects were blocked in mice with parathyroid-specific conditional knockouts (cKO) lacking either α‑Klotho and/or FGFR1-4 [11]. Interestingly, in another study by Canalejo et al., FGF23 decreased PTH production in normal parathyroid glands, but FGF23 did not reduce PTH production in uremic hyperplastic parathyroid glands [12]. The lack of inhibitory effect of FGF23 on PTH production was shown to be due to the decreased expression of FGF23 receptor 1 and the co-receptor Klotho in uremic hyperplastic parathyroid glands. Of note, our patient’s serum creatinine remained normal during burosumab therapy; serum creatinine was 1.01 mg/dL with eGFR of 79 ml/min/1.73 m2 when PTH was at its peak level of 120 pg/mL Moreover, the finding of the temporal increase in serum PTH levels after the initiation of burosumab is consistent with the inhibitory effect of FGF23 on PTH secretion in the setting of tumor-induced osteomalacia. Therefore, in addition to the risks of hypersensitivity reactions and local injection site reactions, secondary hyperparathyroidism is a potential complication of burusumab. Other common adverse reactions of burosumab include back pain, headache, tooth infection, restless leg syndrome, decreased vitamin D level, dizziness, constipation, muscle spasms, and increased phosphorus level [13].

It is well-known that an increase in serum phosphorus levels may promote PTH secretion by the induction of hypocalcemia, inhibition of 1,25-dihydroxy vitamin D synthesis, and direct stimulation of PTH synthesis and secretion [9]. In a large cohort of Chinese patients with TIO, phosphate supplementation was independently associated with the development of hyperparathyroidism, and patients with hyperparathyroidism had lower serum calcium levels compared with patients without hyperparathyroidism [14]. However, our patient had a normal PTH level despite phosphate supplementation prior to treatment with burosumab, and our patient's hypophosphatemia resolved with burosumab without phosphate supplementation. Moreover, our patient's serum calcium levels remained normal during his ongoing burosumab therapy.

Currently, there are no data to guide the treatment of hyperparathyroidism induced by burosumab. Since the increased PTH levels in our patient are accompanied by a rise in the 1,25-dihydroxy vitamin D levels, calcitriol is not an effective therapeutic option for burosumab-induced hyperparathyroidism. Whether calcimimetics are indicated or effective in the treatment of burosumab-induced hyperparathyroidism is unknown at this time. It is also unknown at this time as to when and at what level of PTH treatment of burosumab-induced hyperparathyroidism should be indicated. 

## Conclusions

We reported a 71-year-old patient who presented with significant hypophosphatemia due to tumor-induced osteomalacia. Treatment with burosumab led to the resolution of his refractory hypophosphatemia, but this was complicated by the development of secondary hyperparathyroidism. It has been demonstrated that both PTH expression and secretion were suppressed by FGF23. Since burosumab inhibits the inhibitory effect of FGF23 on PTH, burosumab may result in increased PTH secretion and development of secondary hyperparathyroidism, as is the case in our patient. Further studies are required to define the mechanism and treatment of burosumab-induced hyperparathyroidism.
